# Dynamics of fluvial hydro-sedimentological, nutrient, particulate organic matter and effective particle size responses during the U.K. extreme wet winter of 2019–2020

**DOI:** 10.1016/j.scitotenv.2021.145722

**Published:** 2021-06-20

**Authors:** Hari Ram Upadhayay, Steven J. Granger, Adrian L. Collins

**Affiliations:** Sustainable Agriculture Sciences, Rothamsted Research, North Wyke, Okehampton EX20 2SB

**Keywords:** Laser in situ scattering and transmissometry (LISST), Suspended sediment concentrations, Floc, Hysteresis

## Abstract

The floc size distribution of suspended sediment is a critical driver for in-channel sedimentation and sediment-associated contaminant and nutrient transfer and fate in river catchments. Real-time, in situ, floc size characterisation is possible using available technology but, to date, limited high resolution floc data have been published for fluvial systems draining upland extensive grassland catchments. To that end, suspended sediment floc size distribution and turbidity were characterised at 15-minute intervals using Laser In-Situ Scattering and Transmissometry (LISST) diffraction and a YSI turbidity sonde for six storm events in the upper River Taw (15 km^2^) catchment in SW England. Maximum event discharges (Q) ranged between 4.3 and 20.0 m^3^ s^−1^, with clockwise hysteretic responses (HI = 0.18–0.48) of total suspended solid concentrations (TSS) and Q. The sediment flushing index was highest in the early autumn (0.93) and storm event TSS fluxes varied from 0.04 to 2.9 t km^−2^. This suggests a change in sources or composition of sediment during higher Q and highly variable patterns of sediment flux from event-to-event. The proportion of particulate organic matter (POM) to TSS was highly variable (5–89%) and did not increase with Q, indicating POM source limitation. The fine-grained tail (D_10_ and D_16_) of the floc size distributions decreased during hydrograph rising limbs, with the finest floc sizes associated with the highest TN and TP concentrations at peak Q. The results suggest that dynamic interactions between wet ground and extreme rainfall events can flush significant amounts of sediment from the relatively undisturbed extensive grassland upland catchment. We strongly encourage a sensors-based approach to reveal the spatio-temporal complexity of floc size and associated pollutant export during high Q generated by extreme rainfall since this can help to elucidate processes and mechanisms and generate high-resolution data for water quality modelling without significant user intervention.

## Highlights

• In situ flocs size and turbidity were studied in sequential high discharge events.
• Hydrographs characterised by clockwise hysteresis with a secondary sediment peak.
• Proportion of POM to TSS in the physical samples was highly variable (5–89%).
• Fine (D_10_ and D_16_) floc size diameter was lowest during peak discharges.
• Floc size exerted less influence on TP and TN concentrations.

## Graphical abstract

**Figure:**
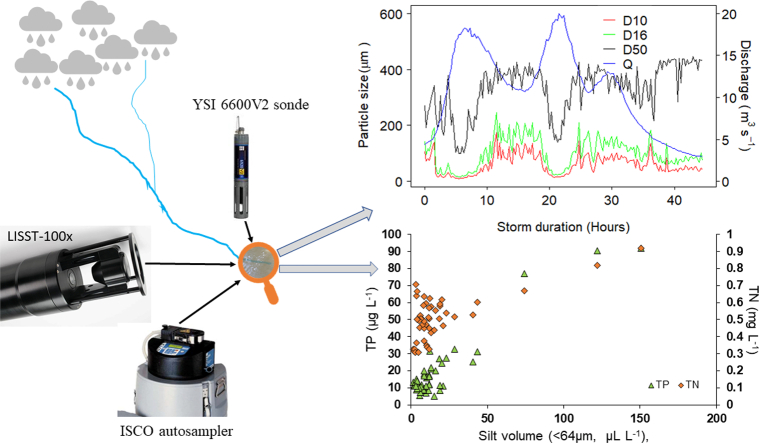

## 1 Introduction

Elevated suspended sediment transport in rivers is a primary environmental and ecological issue around the world (Hauer et al., 2018; Mateo-Sagasta et al., 2018). It plays an important role in contaminant and nutrient transport (Mehta et al., 1989; Luoma et al., 2008; Droppo et al., 2009) and the associated degradation of aquatic habitats and the ecology residing therein (Kemp et al., 2011; Jones et al., 2012; Jones et al., 2014). Reliable quasi-continuous estimates of the concentrations and fluxes of suspended sediment can be assembled using optical turbidity sensors (Walling and Web, 1987; Wass and Leeks, 1999; Walling et al., 2006; Slaets et al., 2014; Ziegler et al., 2014). These sensors are used to help overcome uncertainties and errors associated with regular but infrequent suspended sediment sampling strategies and the concomitant need to use those data in conjunction with discharge measurements and flux estimation algorithms (Webb et al., 1997).

A critical physical parameter governing the behaviour and fate of fine-grained sediment is particle size. Investigations of fluvial suspended sediment have traditionally, focussed on the dispersed mineral fraction of samples to establish the absolute particle size characteristics of transported sediment. In situ however, fine-grained sediment in fluvial systems exists primarily in the form of composite particles known as flocs (Ongley et al., 1981; Phillips and Walling, 1995b; Slattery and Burt, 1997; Droppo et al., 2005; Phillips and Walling, 2005; Grabowski et al., 2011; Krishnappan et al., 2020). Flocs therefore dominate the suspended sediment loads transported by rivers (Walling and Moorehead, 1989; Droppo and Ongley, 1992; Walling and Woodward, 1993; Droppo and Ongley, 1994; Lamb et al., 2020). These flocs are comprised of solid, liquid and gaseous phases, with the former combining mixtures of primary and secondary mineral particles and organic components including living organisms, detritus, faecal pellets and extracellular polymeric substances (Winterwerp and van Kesteren, 2004). Understanding the size characteristics of flocs is particularly important for elucidating the fate of sediment and associated contaminants and nutrients in rivers, lakes and reservoirs, since flocculation has important implications for hydraulic behaviour i.e., the deposition in and dispersal through, fluvial systems (Grangeon et al., 2014; Hoffmann et al., 2020). In these cases, floc size distribution is a crucial factor potentially leading to differing deposition rates in water than the rates for individual particles alone (Zhu et al., 2016; Lamb et al., 2020) with significant impacts on light penetration, contaminant and nutrient concentrations and likelihood of retention on, or in, the river bed (Ahn, 2012; River and Richardson, 2018). Therefore, local floc-size distributions determine the dynamics of suspended sediment fluxes in aquatic systems and associated transport of carbon (C), nutrients and contaminants (Lamb et al., 2020). Despite this important role of flocs, to date, only limited data for the in situ effective particle size (EPS) characteristics of fluvial suspended sediment i.e. flocs have been reported (Williams et al., 2007; Landers and Sturm, 2013; Czuba et al., 2015). Critically where such data do exist, they have not been linked with corresponding estimates of sediment and associated carbon or nutrient exports during extreme weather events. Detailed understanding of EPS at storm event scale remains an important evidence gap for fluvial systems and especially over extended time periods encompassing several storm events (Williams et al., 2007).

Accelerated sediment delivery from the landscape to rivers is partially controlled by storm size (Coynel et al., 2005). Runoff during larger rainfall events strongly connects the catchment area with the river network, thereby facilitating the transfer of high loads of sediment, typically accounting for a significant proportion of the annual fluxes (Oeurng et al., 2010; Zeiger and Hubbart, 2017). Here, riverine particulate organic matter (POM), as a fraction of the sediment, is an important vector for nutrient cycling, since it accounts for up to 20% of total carbon (TC), 60% of total nitrogen (TN) and 90% of total phosphorus (TP) export (Sanchez-Vidal et al., 2013; Bright et al., 2020). Moreover, POM plays a stimulating role for the formation of flocs in various freshwater environments (Guo and He, 2011). However, the export of POM during extreme weather events is unknown for many fluvial systems. Equally, existing floc data for lotic systems do not cover runoff events during hydro-meteorological extremes which are becoming more frequent both in the UK and elsewhere due to climate change. Understanding how extreme precipitation events impact on TN and TP loads in relation to floc size in rivers is crucial for developing more effective and resilient management plans, especially in the context of changing climate (Ockenden et al., 2014).

To investigate the dynamic responses of flocs during storm events, surrogates of suspended sediment such as optical turbidity and laser diffraction metrics can provide in situ sediment concentration and EPS data at high-temporal resolution respectively. Obtaining both sets of information in situ is useful for gaining improved insights into floc size and concentration which, in turn, can provide unique qualitative and/or quantitative information on changing sediment sources and the propensity for rapid transfer or deposition with the rise-to-recession changes of flow during storm runoff events (Landers and Sturm, 2013). The use of optical turbidity as a proxy for particulate C has been reported (Boss et al., 2009) but relatively less is known about the effectiveness of turbidity as a proxy for TN and TP in river systems (Snyder et al., 2018). To address these research gaps, this paper reports recent work investigating the dynamics of storm event suspended sediment export, EPS and sediment-associated C and nutrient transport during storm driven discharge events at catchment scale in the headwaters of the River Taw, southwest England. Within the headwaters of the River Taw catchment, an instrumented landscape observatory has existed since 2018 to monitor discharge and various physico-chemical paraments across nested sub-catchments.

The specific objectives of this study focussed on understanding hydro-sedimentological and associated C and nutrient responses during runoff events in the UK extreme wet winter of 2019–2020, and more specifically, were: (i) to estimate storm event scale suspended sediment and associated C and nutrient export from the headwaters of the upper River Taw observatory; (ii) to examine hysteresis patterns for storm event scale sediment export; (iii) to characterise the EPS of fluvial suspended sediment exported from the headwaters of the upper River Taw observatory, and; (iv) to summarise the insights gained from the new mechanistic data on hydro-sedimentological responses.

## 2 Materials and methods

### 2.1 Study catchment characteristics

**Fig. 1:**
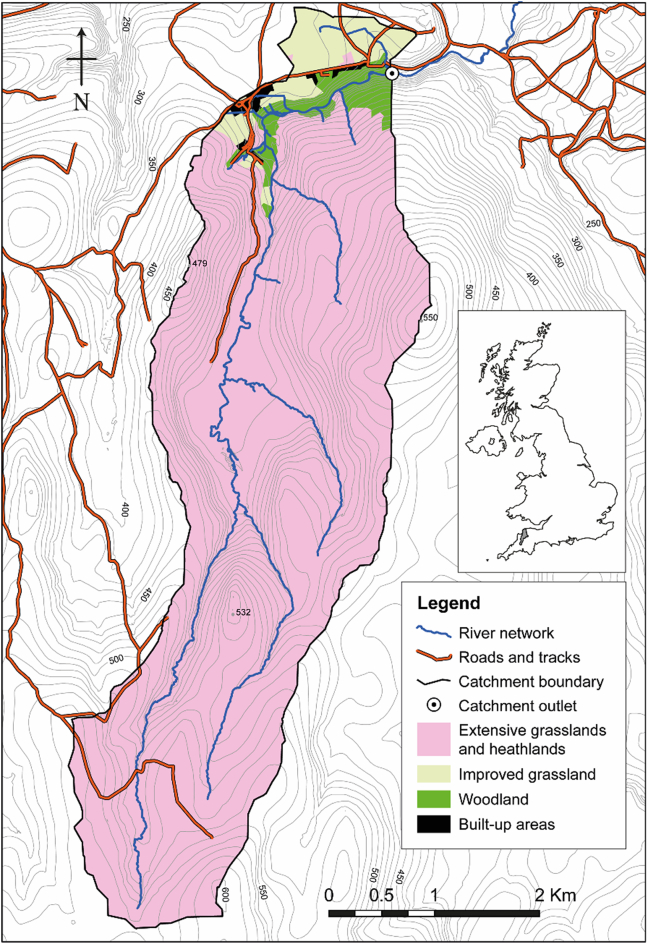
Location and characteristics of the study catchment with the catchment outlet being located at lat: 5072777 N, long: 3.93706 W.

The study was undertaken within the headwaters of the River Taw catchment in Devon, southwest England (Fig. 1). The headwaters of the catchment rise on the Dartmoor granite plateau ca. 550 m above sea level (a.s.l.) and flow northwards for ~10 km to the outlet used for this particular work, located at WGS84 lat: 50.72777 N, long: 3.93706 W, ca. 215 m a.s.l., and draining an area of 15.3 km^2^. The soils on the Dartmoor granite upland consist of peat and podzols and typical annual (1992–2014) precipitation averages 1601 mm on Dartmoor (WGS84 lat: 50.70359 N, long: 3.97760 W), with the majority falling in the winter. The climate is typical of temperate Atlantic Britain (5–20 °C). Approximately 92% of the study catchment area comprises semi-natural marsh, grass and heathlands which support limited numbers of sheep, cattle and ponies managed by so-called less favoured area farms. In the north, around the study catchment outlet, the extensive grassland gives way to a patchwork of more improved grassland (5%), woodland and scrub (3%), and scattered settlements (<1%).

### 2.2 Field deployment of a LISST-100x, multiparameter sonde and autosampler for data collection

The river at the study location flows through a bedrock channel and is predominantly straight. Two stilling wells were fixed to the channel margin to house instrumentation approximately 50 m upstream from an Environment Agency discharge (Q) monitoring station at the catchment outlet (Fig. 1, Fig. S1). One stilling-well housed a LISST-100× (Type-C) (Sequoia Scientific, Inc.) which was deployed in situ during the storm events (Table 1). The particle size information (operating range 2.5–500 μm) is derived from laser diffraction measurements taken in a 5 cm sampling window (Agrawal and Pottsmith, 2000) using an inversion model based on a spherical particle shape assumption. The second stilling well housed a multi-parameter YSI 6600V2 sonde (Xylem Inc., Rye Brook, New York, US) to generate readings for temperature, conductivity, and turbidity. The sensors were calibrated prior to each deployment. Additionally, physical samples were obtained using an ISCO 3700 automatic water sampler (Teledyne ISCO, Lincoln, Nebraska, U.S.).

**Table 1:** Overview of the 6 high Q event monitored on the upper River Taw.

Storm name	Storm event start date	Duration (hours)	Median maximum discharge (m^3^ s^−1^)	LISST scans (n)	Physical samples (n)	Analysed properties on physical samples
	12 Oct, 2019	40.25	3.1 (5.1)	162	15	TSS, POM
	02 Nov, 2019	23.0	5.6 (12.7)	93	6	TSS, POM
	22 Nov, 2019	22.5	3.0 (5.3)	91	13	TSS, POM
Atiyah	10 Dec, 2019	24.5	1.8 (4.3)	99	22	TSS, POM
Ciara	8 Feb, 2020	24	2.45 (8.0)	99	23	TSS, POM, TN, TP
Dennis	15 Feb, 2020	44.5	11.4 (20.0)	179	23	TSS, POM, TN, TP

Instruments were deployed immediately prior to a forecast rainfall event judged to be of sufficient magnitude to cause elevated Q in the river channel (Fig. S2). Instrument internal clocks were reset to a common mobile network synchronised timer device immediately prior to deployment to minimise temporal drift. Sampling intervals for both the LISST-100x and YSI sonde were 15-mins with readings taken on the hour or on 15-min increments thereof. Both devices were set to sample for well in excess of the forecast rainfall duration and corresponding elevated Q event to ensure that each event was covered in entirety. The ISCO autosampler was set to sample at regular intervals, with samples collected at time points which would correspond to those collected by the other instruments, for capturing the duration of the event. As soon as possible after the event, instruments were collected, the data downloaded, and water samples were stored in a refrigerator until laboratory analysis could be undertaken. The 15-minute Q data (m^3^ s^−1^) for the flow gauging station (UK gauging station ID: 50149) were obtained from the Environment Agency.

### 2.3 Sample analyses

Total suspended sediment (TSS) concentrations were determined on physical samples through the change in mass of a pre-weighed GF/C (Whatman, Buckinghamshire, U.K.) filer paper, with a particle retention size of 1.2 μm, following the vacuum filtration of a known volume of sample and subsequent drying at 105 °C (UK Standing Committee of Analysts, 1980). The GF/C filters were burned in a muffle furnace at 500 °C for 30 min to determine the loss on ignition which is considered to provide a proxy of POM.

The TP and TN concentrations in the water samples of two storm events (Table 1) were measures colourimetrically. The TP concentrations were determined through the oxidation of the sample with acidified potassium persulphate in an autoclave at 121 °C and subsequent analysis on an Aquachem 250 (Thermo Fisher Scientific Inc., Massachusetts, U.S.) analyser using a molybdenum blue reaction which has absorbance maxima at 660 and 880 nm (Murphy and Riley, 1962). The TN concentrations were determined through the oxidation of the sample treated with alkaline persulphate in an autoclave at 121 °C to form nitrate. The resulting nitrate was then reduced to nitrite by hydrazine sulphate and total nitrite was also analysed on an Aquachem 250 analyser through the formation of an azo dye with absorbance maximum at 540 nm (Hosomi and Sudo, 1986).

### 2.4 Data analysis

The start of storm Q events was identified based on the occurrence of flow rates that equalled or exceeded the 20% flow exceedance level for base flow. The end of the sampled events was defined as the river flow returning to the initial value or an inflection in the hydrograph associated with the start of another event. Turbidity data was first visually screened to remove obvious errant data and subsequently, to convert turbidity measurements (nephelometric turbidity units, NTU) into TSS (mg L^−1^), a rating curve between TSS and turbidity was developed using 102 samples collected over six storm events to estimate the TSS concentration at unsampled time steps. The median EPS of the individual storms at peak flow was compared to assess the impact of the storm discharge on sediment physical properties. Relationships between discharge, turbidity and EPS (D_60_/D_10_) with TP and TN for high discharge events on the 8 and 15 Feb 2020 were assessed through fitting linear regression.

The discharge ratio (Q_5_:Q_95_) was estimated to assess the flashiness of changes in Q during the individual sampled events (Jordan et al., 2005). To compare the event-based Q - concentration relationships, the Q, TSS, TP and TN data for each individual sampled event were normalized. Based on the normalized data, storm-wise hysteresis index (HI) values were estimated using the method of Lloyd et al. (2016). The HI ranges from −1 to +1 with the sign indicative of the direction of the hysteresis loop. Additionally, the storm flushing index (FI; Vaughan et al., 2017) was estimated to evaluate flushing of sediment or associated nutrients during the sampled Q events as follows:

(1) $$\mathrm{FI}=C_{\mathrm{Qpeak},\mathrm{rising}}-C_{0,\mathrm{rising}}\;$$

where: C_0_ rising, and C_Qpeak_ rising, are the normalized TSS, TP or TN concentrations at the beginning of the event and at the peak flow of the rising limb, respectively. The FI also ranges from −1 to +1, with a negative FI value indicative of a dilution effect associated with the Q event, whereas a positive FI suggests elevated sediment or sediment-associated nutrient concentrations during the Q event compared with baseflow. For each sampled event, TSS and POM loads were calculated using a standard algorithm based on combining concentrations and Q (Nava et al., 2019) (Eq. (2)):

(2) $$L=K\frac{\sum_{i=1}^{n}C_{i}Q_{i}}{\sum_{i=1}^{n}Q_{i}}\bar{Q}\;$$

where: L is the load estimate; $\bar{Q}$ is the average discharge; K is a constant accounting for the duration of the record, and; n is the number of concentration measurements. Additionally, univariate regression was used to assess the relationships between Q, TSS, TN, TP and the D_60_/D_10_ EPS.

## 3 Results and discussion

### 3.1 Hydrological characteristics of the sampled high discharge events

**Fig. 2:**
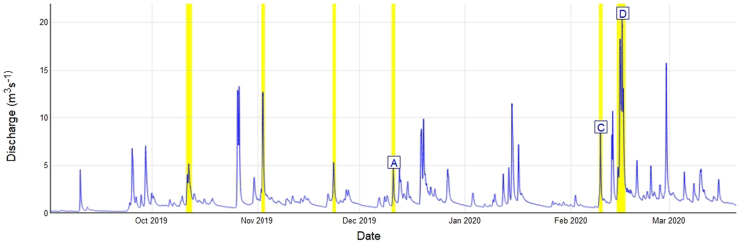
Discharge record for the headwaters (UK gauging station ID: 50149) of the upper River Taw. The focus storms are highlighted by the yellow bars. Letters represent the storm name (A = Ativah, C=Ciara, D=Dennis; see details in Table 1). (For interpretation of the references to colour in this figure legend, the reader is referred to the web version of this article.)

A total of six elevated Q events were sampled over the six-month period (winter of the 2019–2020 water year) (Fig. 2, Table 1). Peak Q during the storm events ranged from 4.3 m^3^ s^−1^ to 20.0 m^3^ s^−1^ with an average elevated discharge duration of 29.7 h. The rising limbs accounted for, on average, ~13 h of time (Table 2). During the extremely wet period of February 2020 a series of large, long-lasting rain events occurred in the study area (Fig. 2). The month of February 2020 was the fifth wettest month ever recorded since 1862 and experienced 237% of the long-term (1981–2010) average February rainfall (Tandon and Schultz, 2020).The catchment was at its wettest condition prior to the event of 14 Feb 2020 (due to storm Dennis) which produced the highest Q (20 m^3^ s^−1^) recorded in this study (Fig. 2 and Table 1). Overall, the UK experienced one of its wettest winters in 2019–2020 and winter rainfall (~470 mm) was 143% greater than the long-term (1981–2010) average. Due to climate change, these extreme weather phenomena are predicted to be more common in the future. Consequently, extreme Q events are predicted to increase in frequency, intensity and duration in the UK (Kendon et al., 2020) and, indeed, around the world. This is likely to have concomitant impacts on long-term riverine TSS concentrations, fluxes and biogeochemical cycles.

**Table 2:** Description of the discharge events based on flow and sediment metrics.

Parameter	Indices	Storm event start date	02 Nov 2019	22 Nov 2019	10 Dec 2019	08 Feb 2020	15 Feb 2020
		12 Oct 2019					
Flow	Hysteresis index	0.31	0.21	0.48	0.34	0.18	0.45
	Flashiness (Q5:Q95)	3.08	5.1	3	4.9	4.97	5.85
	Initial flow (m^3^s^−1^)	0.957	3.7	1.78	0.8	1.44	4.4
	Peak flow (m^3^s^−1^)	5.18	12.7	5.32	4.34	8.05	20
	Event duration (hours)	40.25	23	22	24.5	24.05	44.5
	aMagnitude of flow (%)	441.2	243.2	198.8	442.5	459	354.54
	Average flow (m^3^s^−1^)	3.09	6.61	3.21	2.2	3.27	10.93
	bRelative duration of rising limb (%)	43.4	39.1	31.8	44.9	47.8	48.3
Sediment	Rising duration (hours)	17.5	8	3	8.25	9.5	5.25
	Initial TSS (mg L^−1^)	1.44	12.09	1.44	0.21	4.9	3.67
	Peak TSS (mg L^−1^)	10.61	23.24	9.37	8.13	61.37	76.23
	cMagnitude of TSS (mg L^−1^)	9.17	11.15	7.93	7.92	56.47	72.56
	Flushing index (FI)	0.93	0.1	0.47	0.88	0.36	0.85
	Sediment load (t)	0.94	6.86	1.1	0.55	3.28	44.09
	Specific sediment yield (t km^−2^)	0.06	0.45	0.07	0.04	0.21	2.88
	POM load (t)	0.23	1.75	0.28	0.14	0.83	11.27
	Specific POM yield (t km^−2^)	0.02	0.11	0.02	0.01	0.05	0.74

a The relative differences between the peak flow and initial flow.

b The relative duration of the rising limb compared to the overall storm hydrograph duration.

c The differences between the maximum and minimum TSS concentrations.

### 3.2 Relationships between turbidity and TSS or POM

The monitored turbidity values ranged from 0.1 to 30.3 NTU, with a corresponding median of 0.9 NTU (Fig. S3). Similarly, TSS ranged from 0.7 to 91.4 mg L^−1^ with a median of 2.3 mg L^−1^ while POM varied from 0.1 to 22.7 mg L^−1^ with a corresponding median of 0.7 mg L^−1^. Total suspended sediment concentrations in this study were lower than the TSS concentration observed during winter in the River Creedy, Devon, UK (Walling and Web, 1987). Reasonably linear correlations between turbidity and TSS (TSS = 2.276T - 0.285, R^2^ = 0.9, *p* < 0.001) and turbidity and POM (POM = 0.634T - 0.078, R^2^ = 0.86, p < 0.001) were observed for all storm event physical samples (Fig. S3).

### 3.3 Storm event hydro-sedimentological responses

**Fig. 3:**
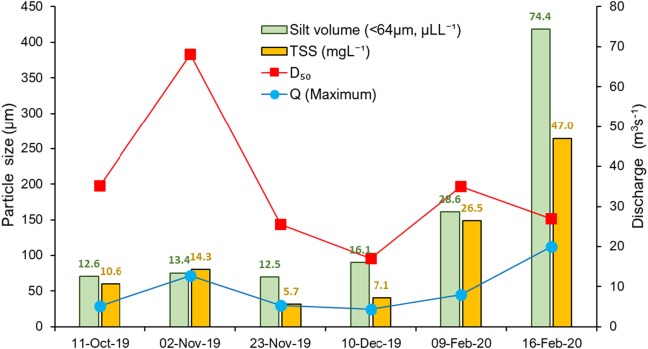
Median effective particle size (EPS) and silt volumes recorded at maximum Q for the six Q events monitored in 2019 and 2020 on the upper River Taw.

The six Q events with their peak Q and corresponding TSS, D_50_ and silt volume are shown in Fig. 3. At peak Q, TSS varied from 5.7 mg L^−1^ to 47.0 mg L^−1^ while silt volume was observed to be ~2.6 times higher in the discharge produced by storm Dennis compared to the previous Q events. The average TSS within the monitored events ranged from 2.1 mg L^−1^ to 18.1 mg L^−1^. The hydrological and sediment transport characteristics of the six monitored Q events are summarised in Table 2.

**Fig. 4:**
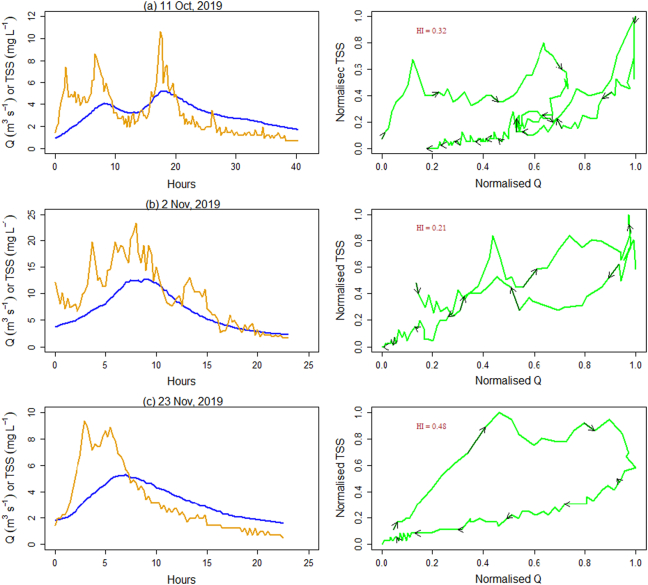

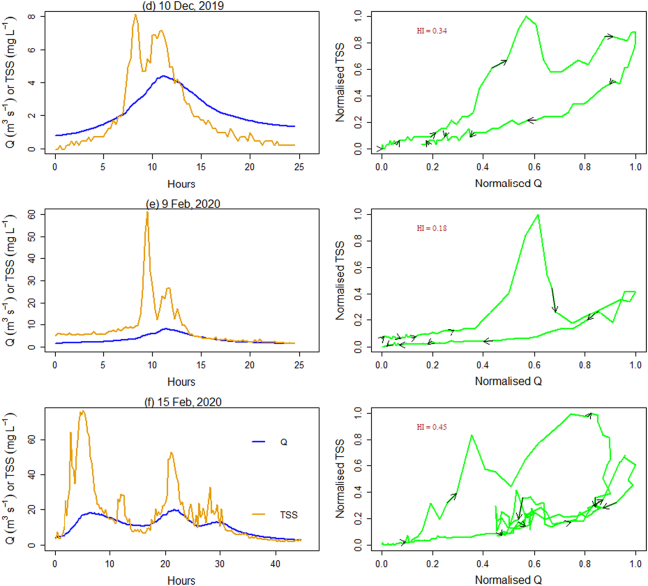
Time series of discharge (blue line) and TSS (orange line), and bivariate scatter plots of normalized Q and TSS for the six Q events monitored on the upper River Taw. Arrows indicate the hysteresis direction through time. (For interpretation of the references to colour in this figure legend, the reader is referred to the web version of this article.)

Bivariate plots show the time series of Q and TSS and their hysteresis for each event (Fig. 4). The Q responses were all relatively ‘flashy’ (Table 2) and occurred in response to effective precipitation. Most of the Q during the storm events were characterised with a secondary TSS peak which did not relate to an increase in Q. The TSS peak in concentrations was consistently observed on the rising limb of the storm hydrographs. The clockwise Q ~ TSS hysteretic loops for all the storm events, with corresponding HI values ranging from 0.18 to 0.48, clearly indicated relatively high contributions from proximal, rather than distal, sources such as in-stream deposited sediments or sediment rapidly mobilised and delivered to the river channel from unpaved tracks which occur in the study catchment (Fig. 1). Although sediment source dynamics cannot be interpreted with accuracy using the HI index alone, sediment source configuration upstream of Q monitoring stations primarily drives hysteresis effects (Marttila and Kløve, 2010; Misset et al., 2019) and clockwise hysteretic loops are indicative of the supply of sediment from the channel bed or highly erodible proximal hillslope sources (Klein, 1984). Sander et al. (2011) further argued that rising limb clockwise hysteretic loops are associated with easily erodible deposited sediment. The multi sediment peaks observed during almost all of the Q events (Fig. 4) are most likely indicative of the complex interplay between various erosion processes and the activation of different sediment sources in addition to hillslopes including channel bank erosion (Smith and Dragovich, 2009). Multi-peak TSS responses can also reflect specific tributary contributions to the main stem TSS (Asselman, 1999), variations in specific source contributions on rising and falling limbs (Vale et al., 2020) as well as the hysteresis effect in the TSS-turbidity relationship (Landers and Sturm, 2013).

High Q can transfer significant amounts of sediment out of river catchments. The highest FI (0.93) was observed in the first monitored Q event of early autumn (11 Oct 2019) despite having a relatively lower peak Q compared to the other storm events (Table 2). This event probably represents the first flush of the 2019 autumn when easily mobilised material was delivered to the river channel following wetting up and the occurrence of saturation-excess surface runoff on local soils. It should be noted that TSS and Q peaks differed between the monitored Q events and this could influence the FI values. Nevertheless, the most extreme Q event associated with storm Dennis (15 Feb 2020) was associated with a high FI (0.85). This event occurred well after the autumn first flush (11 Oct 2019) when the availability of erodible sediment would have recovered and was associated with a significant proportion of the average monthly rainfall for February falling over just 2 days.

The POM as a percentage of TSS was not different across the Q events and remained at ~25%. However, the relative proportion of POM to TSS in the physical samples was highly variable (5–89%) (Fig. S4) and can most likely be attributed to the characteristics of the upland soils having a high proportion of organic matter. The highest variation in the proportion of POM to TSS was observed at Q of ~6 m^3^ s^−1^. The decreasing trend of POM with increasing Q indicated increased mineral soil erosion during high Q (Pawson et al., 2008). At low to medium Q, organic matter rich sediments (e.g., eroded peat soil) might make up a higher proportion of the sediment. The values of % POM observed in this study are within the range reported in existing literature globally (Madej, 2015; Marttila and Klove, 2015; Bright et al., 2020). However, significantly lower concentrations (0.7–3 mg L^−1^) of POM were exported during the low to medium Q compared to the highest Q (19.5 mg L^−1^ POM at 20 m^3^ s^−1^ Q).

This study has demonstrated that extreme Q events are very important for understanding sediment dynamics at catchment scale. Storm event specific TSS yields ranged between 0.04 t km^−2^ and 2.88 t km^−2^ while POM yields ranged between 0.01 t km^−2^ and 0.74 t km^−2^ (Table 2). Most work on TSS yields in the UK (Walling and Web, 1987) reports annual rather than event-specific estimates. The event scale estimates covering a sequence of major winter storms during which most of the annual export would have occurred are however, clearly in agreement with the lowest end of the range (3–286 t km^−2^) of annual yields reported for UK upland catchments with rough grazing previously by Walling et al. (2007). In the case of this study catchment, sediment would seem to be mostly derived from upland organic soils (e.g., plant detritus, peat, soil organic matter) which are sensitive to environmental shocks such as land use (Marshall and Grant, 2017) and antecedent conditions (e.g. wet ground) and extreme rainfall events (e.g., 14 Feb 2020) all of which can significantly influence sediment dynamics within river systems.

### 3.4 Intra-storm variations in suspended sediment EPS

**Fig. 5:**
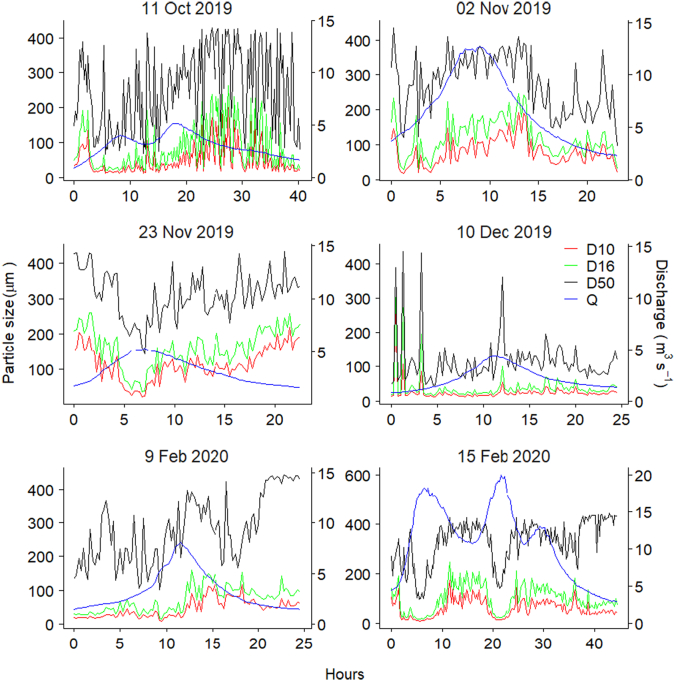
Time-series of discharge and effective particle size (EPS; D_10_, D_16_ and D_50_) for the six Q events monitored on the upper River Taw.

Floc size distribution is important for understanding turbidity signals and the hysteretic behaviour of TSS ~ Q (Landers and Sturm, 2013) since the turbidity signal is more sensitive to fines (<63 μm) than sand-sized (63–125 μm) material (Merten et al., 2014). The median values of D_10_ and D_16_ measured during all Q events ranged between 20 μm to 110 μm and 30 μm to 162 μm, respectively (Fig. 5). The average D_50_ floc size ranged between 95 μm to 382 μm at peak Q (Fig. 3) which is within the range of D_50_ previously measured in the nearby River Exe, southwest UK, by a LISST-100x (Williams et al., 2007). The D_10_ and D_16_ were within the sand size class and may be explained by the catchment geology as well as the formation of larger flocs due to the high POM% in the TSS (Fig. S4) emitted from the peat covered upland drained by the study river.

The temporal variation of floc size in the six high Q events showed that the fine (D_10_ and D_16_) floc fractions decreased to their lowest values during the individual Q events at the corresponding peak Q, with floc diameter increasing again over the falling limbs of the event hydrographs (Fig. 5). A similar relationship between EPS and Q was reported for events sampled on the Yellow River (Landers and Sturm, 2013) and in the Hinkson Creek Watershed (Kellner and Hubbart, 2019) in the USA. In both cases, the authors attributed the intra-storm temporal patterns to changing sources of sediment during the storm hydrographs. Here, entrainment of less cohesive deposited fine sediment during the rising limb of hydrographs may be responsible for the finest floc sizes at peak flows (Sander et al., 2011). Additionally, orthokinetic flocculation processes can decrease floc size during progressively rising Q since shear flow may disaggregate fragile and loose flocs into smaller flocs/primary particles in highly turbulent river systems (Jarvis et al., 2005; Zhu et al., 2016; Byun and Son, 2020). The increased silt size (<64 μm) floc volume with Q (Fig. 3) further supports our interpretation that disaggregation of large flocs during rising Q affects the EPS distribution in the study river. A common explanation for flocs moving during the rising limb and peak Q of storm runoff hydrographs is that the bulk of the TSS most likely originates from in-channel sources (e.g., previously deposited sediment) whereas TSS transported on the hydrograph falling limb is more likely to be associated with hill slope or more distal sources (Lawler et al., 2006). The drawdown effect of the falling limb of hydrographs can erode exposed channel banks, but within our study area, well developed heavily eroding channel banks are not common. Such features become more prevalent further down the catchment system. Although it is often hypothesized that smaller flocs may not be stored on and within the stream bed between stormflows since low stream velocities can readily mobilise and transport them (Landers and Sturm, 2013), this can be challenged. Indeed, our timeseries of floc size data on the 15 Feb 2020 (Storm Dennis) suggests that this pattern is not universal and that fine flocs can quickly settle onto the stream bed even in our flashy study catchment. The time series of TSS concentrations measured in the physical samples for Storm Dennis exhibits an increase with increasing Q (Section 3.4) which independently supports our interpretation of the deposition of fine flocs on the streambed between stormflows which is subsequently remobilised as Q increases during storm events. Worrall et al. (2018) also reported that intra-storm deposition is prevalent in the upper reaches of the River Trent, UK. The EPS data also indicated that the D_60_/D_10_ ratio had a slight effect on the variance in turbidity during the highest monitored Q events (e.g., Storms Ciara and Dennis; Table S1, Fig. S5) which might be attributed to the flux of larger floc sizes. Nevertheless, it has been noted that smaller size flocs affect turbidity and frequently explain the hysteresis in turbidity-TSS (Landers and Sturm, 2013).

### 3.5 Insights gained from, and implications of, high-resolution floc size measurements for nutrient export

**Fig. 6:**
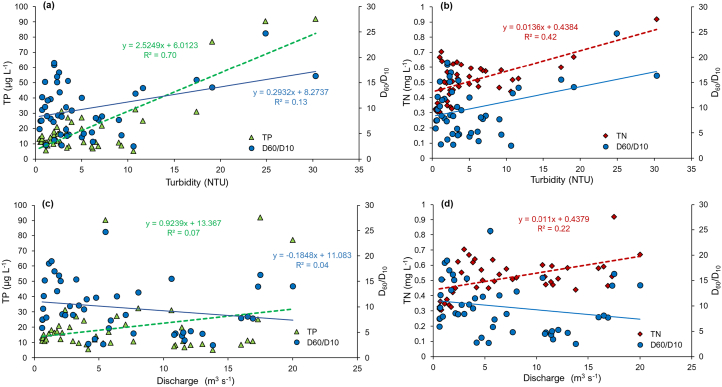
Relationships between Q, turbidity and effective particle size (EPS; D_60_/D_10_) with TP (a, c) and TN (b, d) during two of the Q events monitored (8 and 15 Feb 2020) on the upper River Taw.

**Fig. 7:**
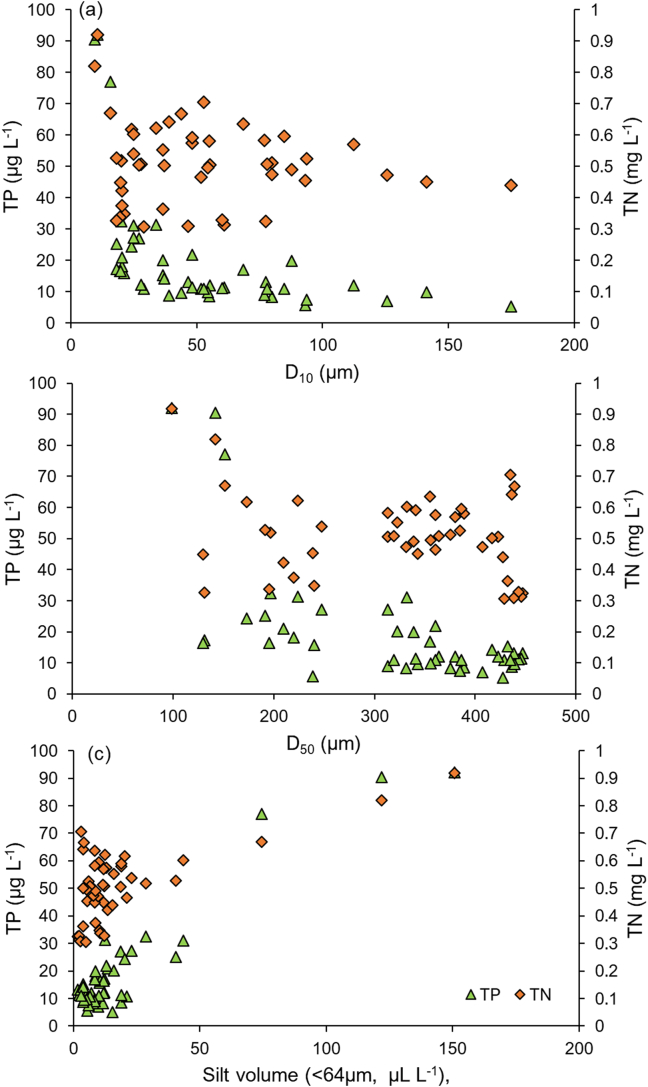
Dependency of TP and TN on floc size: (a) D_10_; (b) D_50_, and; (c) silt volume, in the upper River Taw.

The concentrations of TP and TN were measured in the physical samples collected in the high Q events associated with two of the sampled storms; namely Storms Ciara and Dennis (Table 1). The Q, turbidity and flocs size distributions for these storms in relation to TP and TN concentration are illustrated in Fig. 6, Fig. 7. As Fig. 6 a and b show, the TP and TN contents increased above a turbidity of 10 NTU during these high Q events but were only weakly related to the storm Q. The correlation coefficients for TP and TN with turbidity were 0.83 (*p* < 0.001) and 0.65 (p < 0.001), respectively. The high-resolution time-series data demonstrated that extreme events affect nutrient delivery even from an upland extensive grassland landscape with the FI increasing by +0.45 and by +0.82 for TN and TP, respectively, for Storm Dennis, the largest storm event sampled by the field campaign (Fig. S6). The relationship between TN and turbidity reported here is in good agreement with the correlation coefficients reported by Snyder et al. (2018) for catchments with a range of land use types. The strong positive relationships between turbidity and TP and TN suggest that turbidity values can be an effective proxy for TP and TN despite the numerous complicating factors (e.g., floc size, shape) that are known to affect turbidity measurements. Although, relatively little is known about the utility of turbidity measurements for the prediction of TP and TN in undisturbed landscapes, Villa et al. (2019) suggest that turbidity readings can serve as useful proxies for TP in catchments with a substantial percentage of intensive agricultural land. Interestingly, the floc size exerted less influence in this study catchment on TP and TN concentrations since these remained between 10 and 30 μg L^−1^ and 0.3–0.7 mg L^−1^ respectively, regardless of variations in EPS (Fig. 7 a, b). The relation between floc size and nutrient concentrations is not typically quantified at the catchment scale, and it therefore remains challenging to make direct comparisons with the data from other catchments. However, the TP and TN concentrations increased with increasing silt (<64 μm) volume beyond 50 μL L^−1^ (Fig. 7c) which can be attributed to the well-documented control of fines on sediment-associated nutrient concentrations. Here, the control exerted by changing sediment sources with very fine grain size fractions (<25 μm) dominated by clay minerals and organics should be borne in mind (Fig. S5 c, d). Overall, the Q event associated with Storm Dennis exported 0.62 kg TN ha^−1^ (0.062 t km^−2^) and 0.025 kg TP ha^−1^ (0.0025 t km^−2^) from this catchment which is comparable to the TN and TP export coefficients estimated for rough grazing grassland in the Slapton catchment, South Dartmoor, UK (Johnes, 1996). However, this single extreme event (Fig. 4f) represented only 0.5% of time in a year but exported about 11% of TN and TP out of the catchment compared to the River Taw long-term annual average load (1990–2000) at our monitoring location (Williams and Newman, 2006). Further research is needed in agricultural catchments with various levels of N and P in their soils to understand the impact of floc sizes on N and P fluxes in river systems.

Flocculation can significantly alter sediment settling patterns and thereby the dispersal patterns of nutrients and contaminants through river systems, and floc size, shape and structure can vary both in time and space (Jarvis et al., 2005; Lamb et al., 2020). Sensor-based approaches better capture flocs characteristics and associated concentrations across the full range of flow regimes and at a time scale where concentration and discharge vary (Fazekas et al., 2020). Here, the physical collection of water samples from fluvial environments and transportation to the laboratory for EPS analysis cannot be assumed to provide reliable data on the natural EPS distribution of flocculated fine sediment. This is because physical sampling significantly changes both physical hydrodynamic forcing and biological activities which are responsible for floc formation in natural systems (Phillips and Walling, 1995a; Jarvis et al., 2005). It is therefore more meaningful to deploy non-destructive techniques *in- situ* within the fluvial environment for more accurately assessing weather-driven variation in water quality and the effectiveness of agricultural pollution reduction schemes at large spatio-temporal scales.

### 3.6 Limitations of the work

The reliability of the data using the LISST portable laser device has been reported by several studies using comparisons with alternative sediment analysis techniques (Gartner et al., 2001; Serra et al., 2001; Mikkelsen et al., 2005; Mikkelsen et al., 2006; Czuba et al., 2015). Nonetheless, the new data on EPS and sediment-associated nutrient transport during storm events of contrasting magnitude reported in this paper should be interpreted in the context of some challenges and limitations. It is important to acknowledge that all particle size data are operationally-defined (Agrawal and Pottsmith, 2000). This study assumes the presence of spherical particles, but no assumptions are made regarding particle density or mass. A certain degree of flow disruption in conjunction with field deployment of the LISST-100× is unavoidable. Here, the potential for some fragmentation of flocs due to changes in shear stress should be considered (Gibbs, 1981; Gibbs and Konwar, 1982). Furthermore, high external light can introduce particle sizing inaccuracies (Reynolds et al., 2010) and in-channel deployment of the LISST-100× can result in the trapping of leaves and other small debris in the area of the laser, or indeed the sensor used for turbidity monitoring although these issues can be minimised through the use of vertical stilling wells as deployed in this study. Stilling wells must be attached securely to the straight section of the stream bank to avoid sensor loss during extreme Q events.

## 4 Conclusions

Based on the field deployment of state-of-the-art instruments during six storm Q events of the 2019–2020 extreme wet winter in the upper River Taw, high-frequency real time TSS concentrations and export, floc size distributions and sediment-associated nutrient (N and P) and POM fluxes were examined. The high Q events significantly increased median TSS and the silt (<64 μm) volume concentration which suggests that these events can transfer considerable amounts of sediments out of upland extensive grassland catchments. The Q, TSS and POM concentrations likely impacted the formation of flocs. Increasing floc diameter during the falling limbs of the storm hydrographs despite the TSS peak being observed before peak Q provide evidence that low-frequency sampling regimes are insufficient to describe the hydro-sedimentological dynamics in river systems. Discharge-driven variation in floc size distributions is of immediate value for developing and testing sediment transport models to assess sediment dynamics and nutrient and contaminant transport in river systems. This study provides new information on EPS distributions and TSS, POM and nutrient fluxes in a high energy river system during the high magnitude storm events of the extreme wet winter of 2019–2020 which are projected to be more frequent in the future under climate change scenarios. It is of great importance to monitor continuously extreme Q events in different catchment settings to understand the impact of such events on aquatic systems. In the context of climate and land use change, achieving the ambitious 2030 UN agenda for Sustainable Development Goals related to improving water quality by reducing pollution requires enormous efforts. Here, the high-resolution and accuracy offered by deploying sensors in situ, makes it possible to capture the variability in river Q and episodically-transported pollutants during large flood events irrespective of river size and site accessibility both in developed and developing countries.

## Author contribution

All authors listed have made a substantial, direct, and intellectual contribution to this article, and approved it for publication.

## Declaration of competing interest

The authors declare that there are no known competing financial or personal interests that can influence the data and interpretation of this paper.

## Appendix A Supplementary data

Supplementary material Image 1
