# Supplementary material for: Dynamics of fluvial hydro-sedimentological, nutrient, particulate organic matter and effective particle size responses during the U.K. extreme wet winter of 2019–2020
Source: Sci Total Environ. 2021 Jun 20;774:145722. doi: 10.1016/j.scitotenv.2021.145722 (PMC8073540; doi:10.1016/j.scitotenv.2021.145722)
Supplement: Supplementary file 1 — Supplementary material [file mmc1.docx]

Supplementary Information

**Dynamics of fluvial hydro-sedimentological, nutrient, particulate organic matter and effective particle size responses during the U.K. extreme wet winter of 2019-2020**

Hari Ram Upadhayay*, Steven J. Granger, Adrian L. Collins

*Sustainable Agriculture Sciences, Rothamsted Research, North Wyke, Okehampton EX20 2SB*

[*hari.upadhayay@Rothamsted.ac.uk](mailto:*hari.upadhayay@Rothamsted.ac.uk)

| 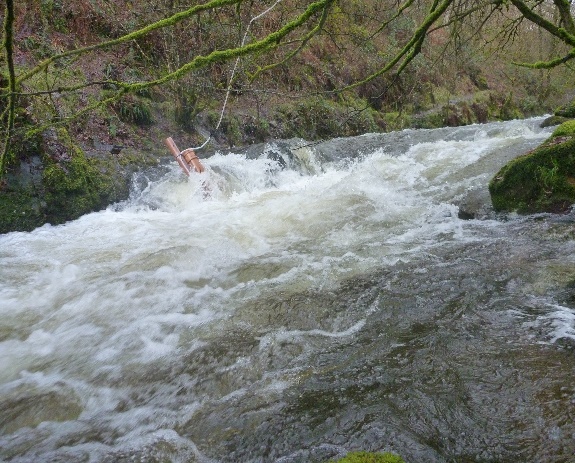  **(a)** | 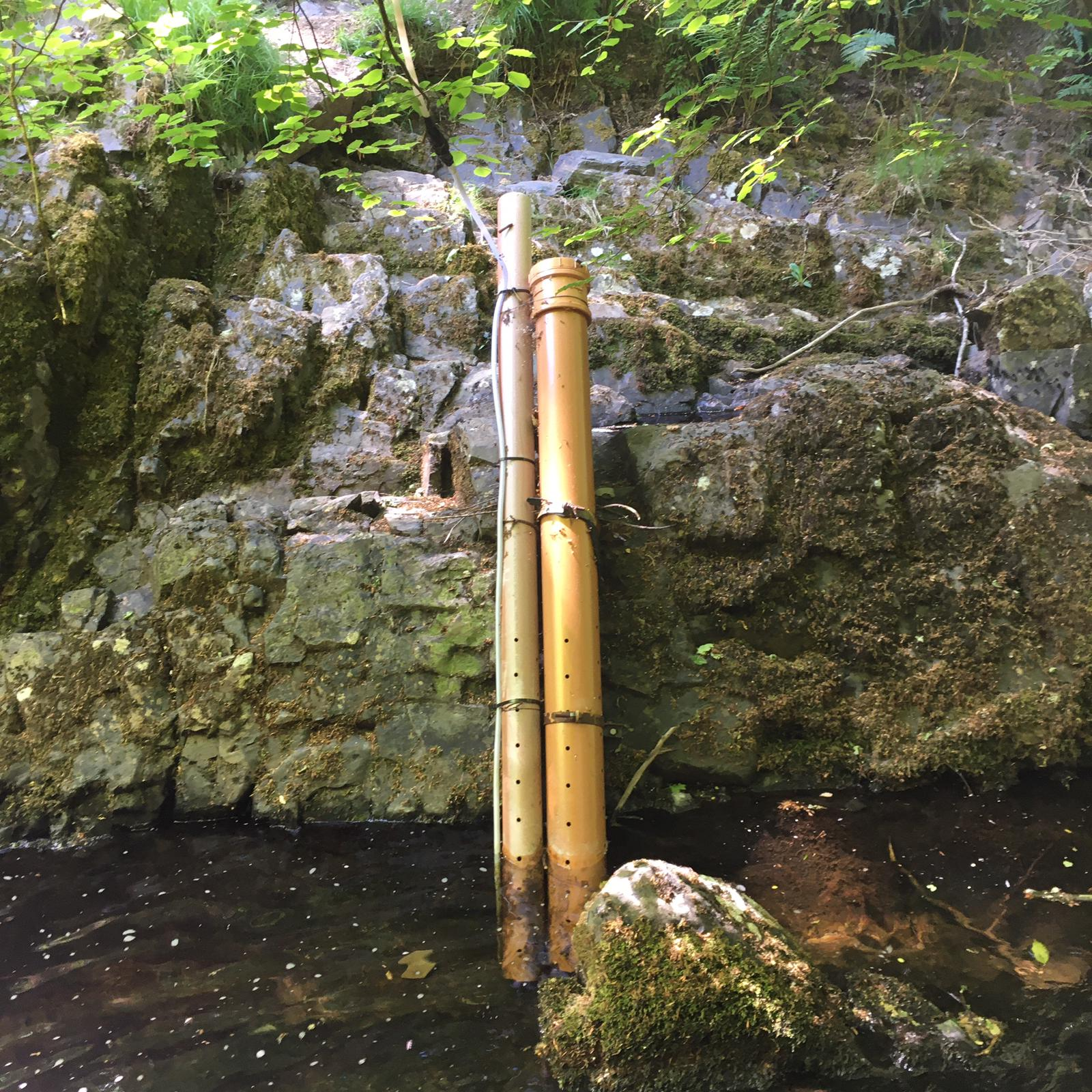  **(b)** |
| --- | --- |

Fig. S1. Field deployment of a LISST-100x and multi-parameter YSI 6600V2 sonde (a) during high flow (e.g.14 Feb 2020) and (b) at base flow (30 Sep 2019). The larger stilling well was used to support the inlet pipe for an automatic ISCO 3700 water sampler.


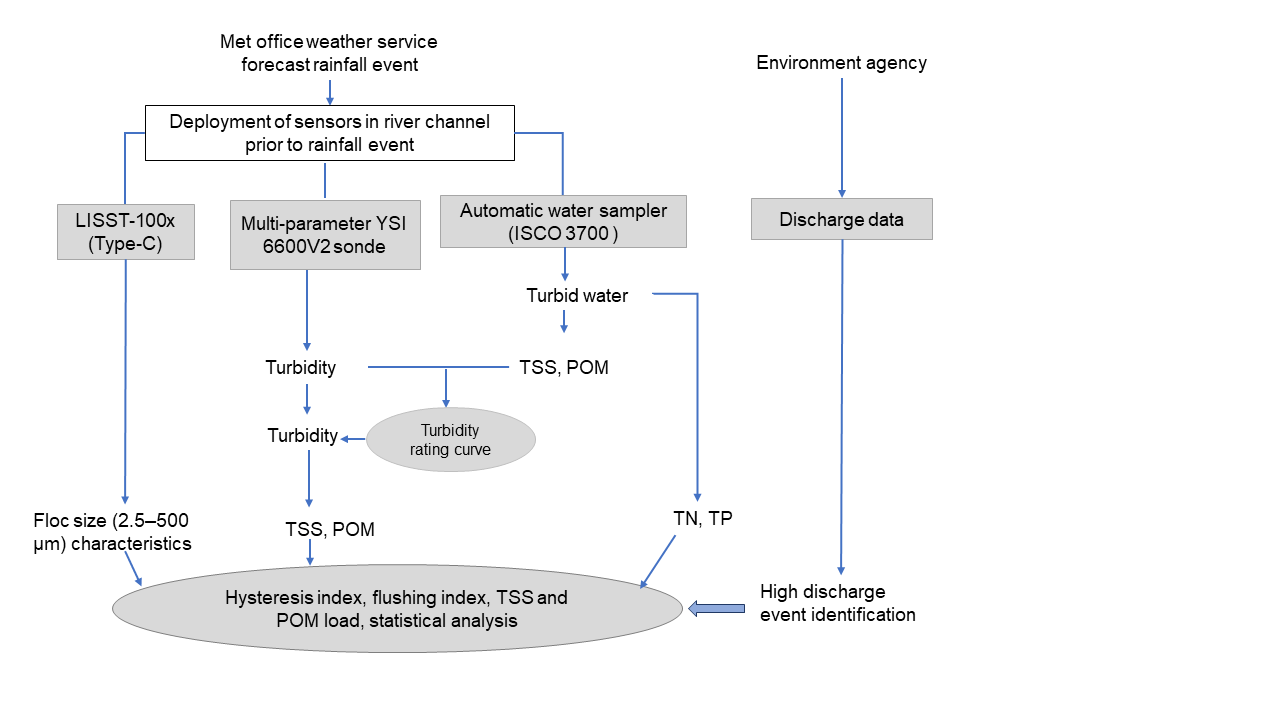


Fig. S2 Flow chart of the research methodology.


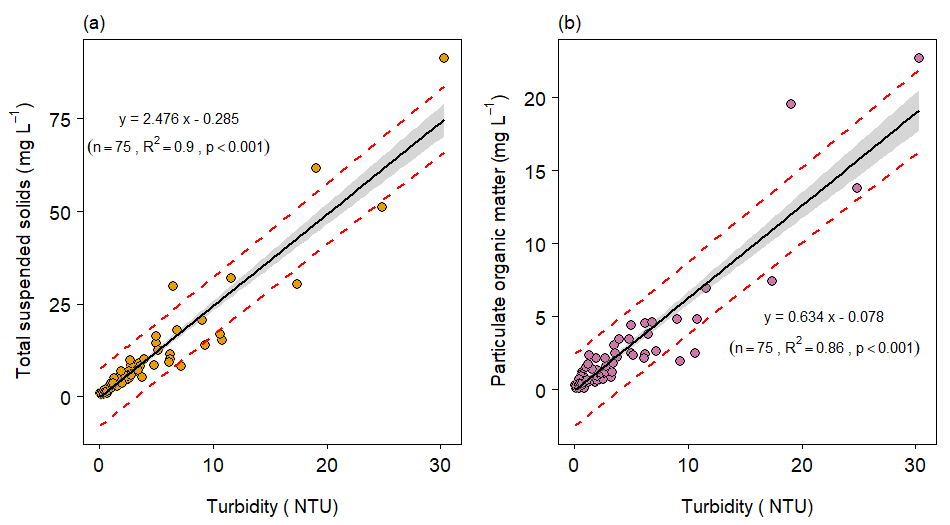


Fig. S3 Relationships between (a) turbidity and TSS, and (b) turbidity and POM in the physical samples collected during discharge events on the upper River Taw. Grey shaded areas represent the 95% confidence intervals. The red dashed lines depict the 95% prediction intervals.


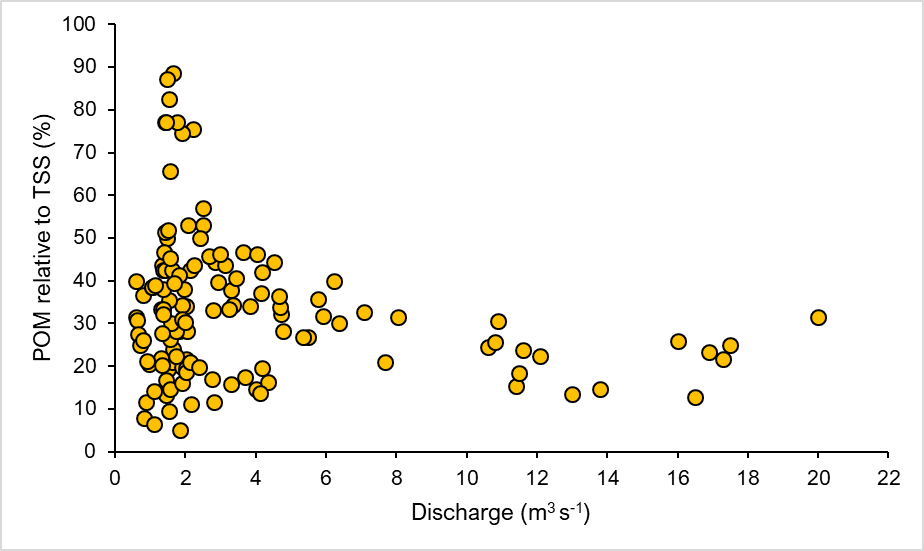


Fig. S4 POM as a percentage of TSS in the physical samples.


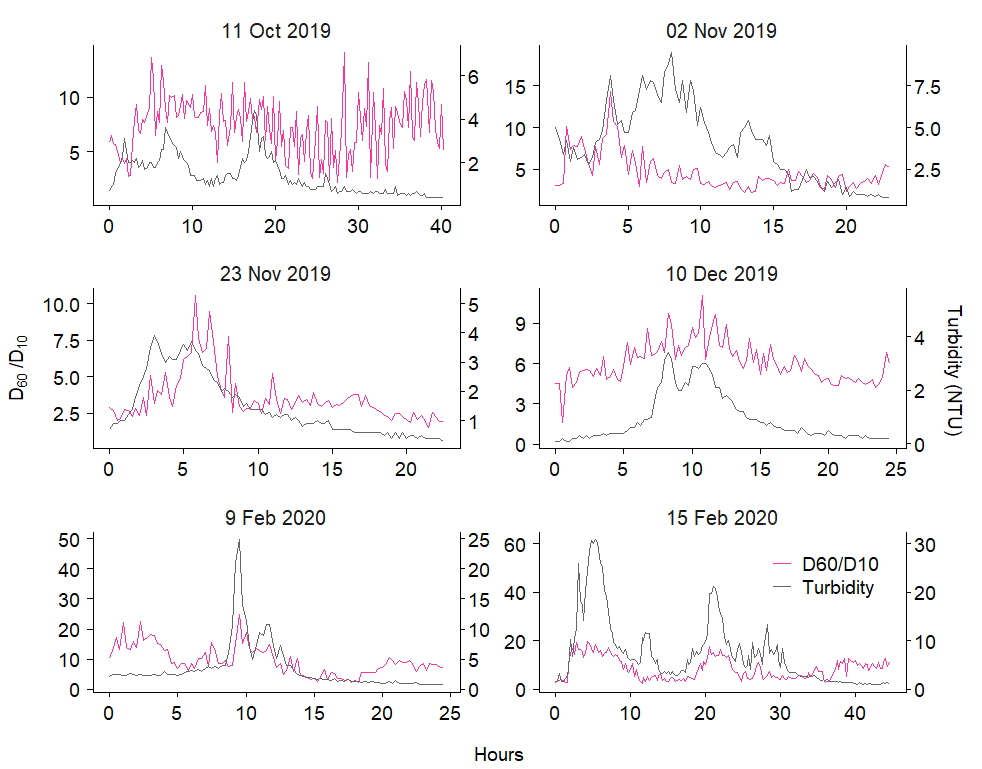


Fig. S5 Time series of turbidity and EPS (expressed as the D_60_/D_10_ ratio) in the upper River Taw.


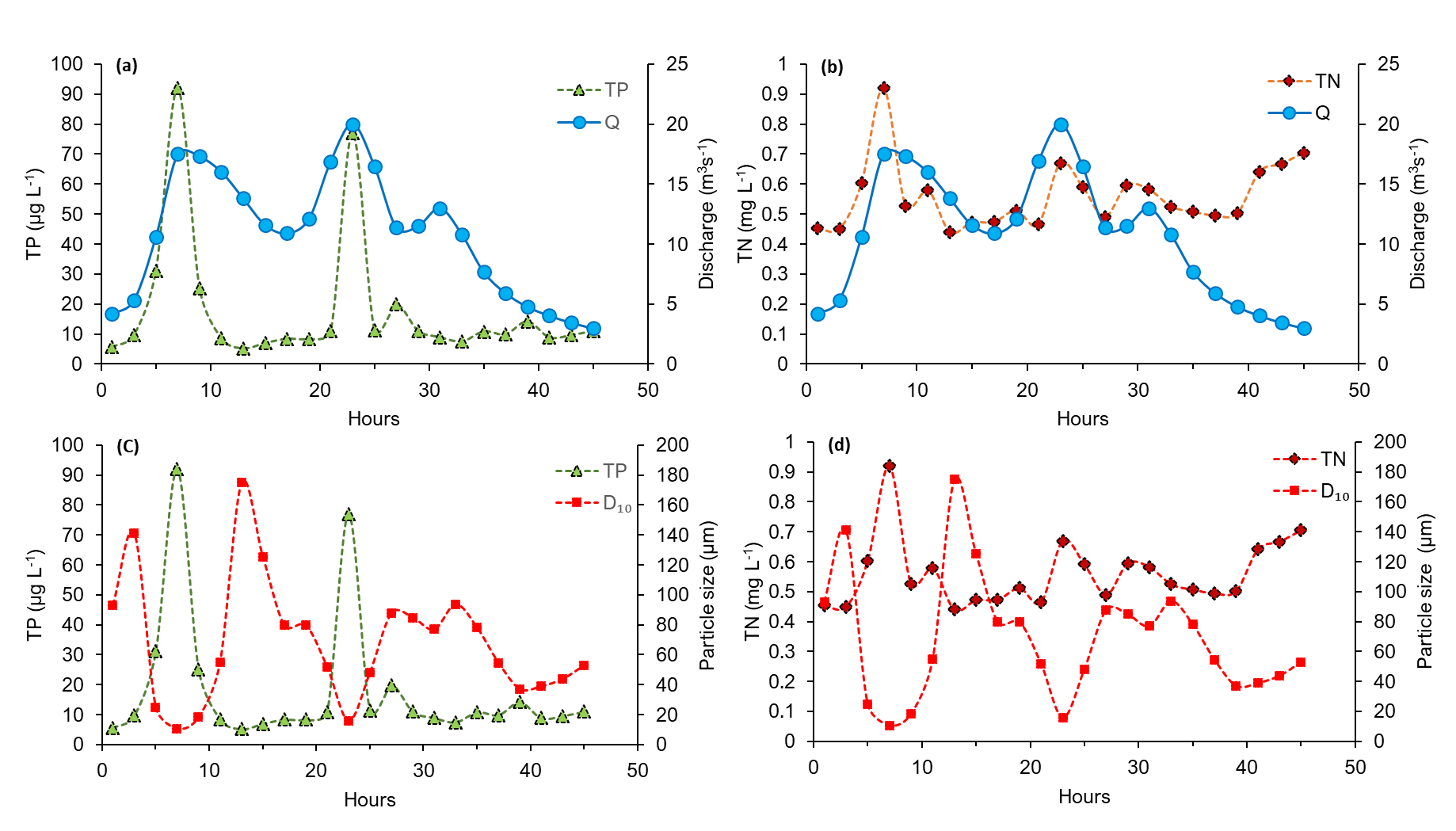


Fig. S6 Dynamics of discharge and EPS (D_10_) with: (a) TP and (b) TN for the high discharge event during 15–17 Feb 2020 in the upper River Taw.

Table S1 Results of regressions between log-transformed D_60_/D_10_ and turbidity in the upper River Taw.

| Storm event start date | Average D_60_/D_10_ | Average turbidity (NTU) | Slope | R^2^ | p-value |
| --- | --- | --- | --- | --- | --- |
| 12 Oct 2019 | 7.43 | 1.4 | 0.07 | 0.01 | 0.13 |
| 02 Nov 2019 | 4.64 | 4.0 | 0.14 | 0.06 | 0.01 |
| 22 Nov 2019 | 3.56 | 1.5 | 0.37 | 0.47 | <0.001 |
| 10 Dec 2019 | 6.06 | 0.9 | 0.19 | 0.54 | <0.001 |
| 8 Feb 2020 | 9.49 | 3.4 | 0.30 | 0.20 | <0.001 |
| 15 Feb 2020 | 8.47 | 7.4 | 0.19 | 0.11 | <0.001 |
